# Nectar Yeasts in the Tall Larkspur *Delphinium barbeyi* (Ranunculaceae) and Effects on Components of Pollinator Foraging Behavior

**DOI:** 10.1371/journal.pone.0108214

**Published:** 2014-10-01

**Authors:** Robert N. Schaeffer, Cody R. Phillips, M. Catherine Duryea, Jonathan Andicoechea, Rebecca E. Irwin

**Affiliations:** 1 Department of Biological Sciences, Dartmouth College, Hanover, New Hampshire, United States of America; 2 Rocky Mountain Biological Laboratory, Gothic, Colorado, United States of America; Central China Normal University, China

## Abstract

Microorganisms frequently colonize the nectar of angiosperm species. Though capable of altering a suite of traits important for pollinator attraction, few studies exist that test the degree to which they mediate pollinator foraging behavior. The objective of our study was to fill this gap by assessing the abundance and diversity of yeasts associated with the perennial larkspur *Delphinium barbeyi* (Ranunculaceae) and testing whether their presence affected components of pollinator foraging behavior. Yeasts frequently colonized *D. barbeyi* nectar, populating 54–77% of flowers examined depending on site. Though common, the yeast community was species-poor, represented by a single species, *Metschnikowia reukaufii*. Female-phase flowers of *D. barbeyi* were more likely to have higher densities of yeasts in comparison to male-phase flowers. Pollinators were likely vectors of yeasts, as virgin (unvisited) flowers rarely contained yeasts compared to flowers open to pollinator visitation, which were frequently colonized. Finally, pollinators responded positively to the presence of yeasts. *Bombus* foragers both visited and probed more flowers inoculated with yeasts in comparison to uninoculated controls. Taken together, our results suggest that variation in the occurrence and density of nectar-inhabiting yeasts have the potential to alter components of pollinator foraging behavior linked to pollen transfer and plant fitness.

## Introduction

Microbial organisms abound in natural systems. For plants, processes as critical as nutrient acquisition and defense against herbivores can be fueled and enhanced by plant interactions with microbes [1]–[3] and almost all plant parts examined interact with microorganisms, including roots, leaves, and flowers. Even floral nectar hosts a microbial community, including yeasts, bacteria, filamentous fungi, and smuts [4]–[8]. Even though the chemical composition of nectar could strongly select against microorganisms [9]–[11], recent studies suggest that microorganisms routinely colonize the nectar of many plant species [5], [8], [12]. For example, community-level surveys suggest that as many as 95% of plant species in a community can harbor yeasts in their nectar [8], [12], [13]. Despite the growing number of studies documenting the proportions of plant species harboring microbes in their nectar and nectar microbial community composition, few studies have dissected spatial and temporal dynamics of nectar microbial occurrence and density in single plant species [12], [14], [15]. This limited number of studies has observed significant variation in microbial communities among flowers within plants, plants within populations, and among populations. Understanding the magnitude of such variation could have important implications for how plants interact with their pollinators [12], with subsequent effects on pollen flow, plant fitness, and patterns of pollinator-mediated natural selection.

Floral nectar is a critical resource meditating interactions between plants and animals [16], and nectar-inhabiting microorganisms have the potential to modify a suite of traits important for pollinator attraction [6], [17], [18]. For example, yeast activity in nectar can drastically reduce sugar concentrations and alter sugar ratios [6], [13]. Moreover, yeast metabolic activity can affect other floral attractive traits, such as temperature [19], scent [17], [20], and amino acid content [21], [22]. Indeed, evidence suggests that yeasts can affect pollinator foraging behavior with consequences for plant fitness [23]–[25]. For example, high densities of yeast in the nectar of the low larkspur *Delphinium nuttallianum* increased the amount of nectar removed by bumble bee and hummingbird pollinators relative to flowers with low yeast densities [25], a pattern similar to that found for the bumble bee pollinated *Helleborus foetidus* [24]. However, these beneficial effects of yeasts on pollinator foraging are not universal [18], [26]. More studies are needed that link the presence and activity of nectar-inhabiting yeasts to plant-pollinator interactions to assess the generality of these findings.

Here, we investigated variation in the occurrence and density of nectar-inhabiting yeasts of the montane tall larkspur *Delphinium barbeyi* (Ranunculaceae; hereafter *Delphinium*), and their effects on pollinator foraging behavior. Specifically, we investigated the (1) the frequency of occurrence, density, and identity of yeasts in the floral nectar of *Delphinium*; (2) how their dynamics vary spatially and with flower sex; and (3) the relationship between yeast presence and potential changes in pollinator foraging behavior. We tested the following three predictions. First, though self-compatible, *Delphinium* relies on a suite of pollinators for successful reproduction through out-crossing [27]. Recent studies have documented that such pollinators can serve as vectors of yeast in other plant-pollinator systems [12], [14], [28]. Thus, we predicted to find similar vectoring in *Delphinium*. Second, assuming pollinators disperse nectar-inhabiting yeasts and because pollinator visitation can vary among sites as well as between male and female phases of flowers [29]–[31], we predicted that nectar yeast densities would also vary among *Delphinium* populations and between floral sexual phases. Third, the foraging behavior of the dominant pollinators of *Delphinium* (bumble bees) are sensitive to changes in nectar traits, such as nectar volume, sugar concentration, sugar ratios, and scent [32], [33], many of which may change as a function of yeast presence or metabolic activity [6], [18]. Thus, we predicted that yeasts would alter bumble bee pollinator foraging behavior. Given that pollinators are important for *Delphinium* reproduction [34], any potential changes in pollinator foraging behavior could alter plant fitness.

## Materials and Methods

### Ethics statement

This research was conducted in accordance with the recommendations and approval of the Rocky Mountain Biological Laboratory (RMBL) Research Committee and is in compliance with a special research permit issued to them by the US Forest Service.

### Study system

We conducted this study using the tall larkspur, *Delphinium barbeyi*, near RMBL in Gothic, Colorado, USA (elevation: 2891 m). Around RMBL, *D. barbeyi* is a long-lived, perennial herb that blooms from mid-July to late August. Commonly found in wet meadows in subalpine and montane regions of Wyoming, Utah, and Colorado, USA [35], *D. barbeyi* typically grows multiple stalks (∼1.5 m in height) that produce tens to hundreds of hermaphroditic, protandrous flowers [36]–[38]. The flowers of *D. barbeyi* have two nectar spurs contained within the fused upper petals with a nectar standing crop of 1.8+0.05 µl per flower in the morning before pollinator visitation and a sugar concentration of 44+3% (Irwin, *unpub data*). The most common pollinators of *D. barbeyi* around RMBL are bumble bees (especially *Bombus appositus* and *B. flavifrons*) but flowers are also visited by hummingbirds (*Selasphorus platycercus* and *S. rufus*), hawkmoths (*Hyles lineata*), and small bees and flies [36], [39]–[41].

### Nectar sampling

Nectar sampling was conducted at three study sites near RMBL: the 401-Trailhead Meadow (Lat, Long: 38.965, −106.988), Marriage Meadow (Lat, Long: 38.967, −106.99), and the Beaver Dam Meadow (Lat, Long: 38.974, −106.993). To assess both the frequency of yeast occurrence and their density in *D. barbeyi*, floral nectar was collected in summer 2013 using the following approach. Whole stalks on individual plants were bagged using fine-mesh bags constructed of bridal veil to prevent flower visitation by pollinators or other insects and allow for nectar accumulation. On each stalk, we took care to mark whether flowers were open or virgin (enlarged bud) prior to bagging, in addition to their sexual phase. Flowers were scored for their sex as either male (pollen-dehiscing anthers) or female (dried anthers and stigmas were reflexed). After 48 hrs, we returned to bagged stalks and collected marked flowers (*N* = 2–25 flowers per stalk). Flowers were stored individually in Zip-loc bags, placed in a cooler, and returned to the laboratory for processing of nectar samples. All processing occurred within 12 hours of collection. Sites were sampled twice between 12 July and 4 August 2013, and 10 stalks (each on a different plant) were bagged per sampling event per site.

### Yeast density, and identification

To determine yeast presence and density, we extracted nectar from flowers with calibrated microcapillary tubes and recorded the volume of each sample. Each sample was diluted with 9 µl of 30% lactophenol cotton blue solution to facilitate microscopic visualization of yeasts. Cell density (cells/mm^3^ of nectar) was determined using a Neubauer chamber (Hausser Scientific, Horsham, PA, USA) and standard cell counting methods at a magnification of 400x (Nikon Eclipse E400, Melville, NY, USA) [6], [12]. We identified cells as yeasts based on size and morphological features, such as the presence of budding cells and large vacuoles, which contain refractive corpuscles. These density estimates thus integrate across all potential yeast taxa that were present in a sample, since this morphology-based identification cannot distinguish among different yeast species. This coarse level of identification however was suitable to assess yeast occurrence and density and has been successfully used in other yeast surveys [6], [12].

Yeast identity was determined by removing nectar from a subset of *Delphinium* flowers (*N* = 28 flowers, each from a different plant) and streaking samples onto yeast malt (YM) agar (Catolog number: Y3127, Sigma-Aldrich, Inc., Saint Louis, MO, USA) supplemented with chloramphenicol. Including chloramphenicol inhibited bacterial growth so that we could focus on yeasts; assessing nectar bacterial abundance and diversity was beyond the scope of this study but can be assessed in future research. For each plate, one colony was picked for each morphologically distinct colony type and subsequently plated for further purification of isolates. DNA from isolates was extracted and amplified using a Sigma REDExtract-N-Amp Tissue polymerase chain reaction (PCR) kit (Sigma-Aldrich, Inc., Saint Louis, MO, USA). PCR was performed in a volume of 20 µl using 0.8 µl of extracted DNA, 10 µl of REDExtract-N-Amp PCR Reaction Mix, 0.15 µl of each primer at 50 µM and 8.9 µl of H_2_O. We amplified a region of the D1/D2 domains of the large subunit nuclear ribosomal RNA gene using the primers NL1 and NL4 [42]. PCR amplification was performed using a DNAEngine Thermal Cycler (Bio Rad Laboratories, Hercules, CA) using a touchdown PCR protocol outlined in Belisle et al. [14]. PCR products were separated by gel electrophoresis using 2% tris-borate-EDTA (TBE) agarose gel and visualized using ethidium bromide staining and subsequent UV transillumination (UVP Biosystems, Upland, CA). Samples that produced a visible band were purified by addition of 2 µl of ExoSAP-IT (Affymetrix, Santa Clara, CA) and incubation at 37°C for 30 mins followed by an inactivation step at 80°C for 15 mins. Purified products were sequenced on an ABI3730 Genetic Analyzer (Applied Biosystems, Carlsbad, CA) in one direction using the primer NL1. We trimmed and edited all sequences by eye using the program Sequencher v4.6 (Gene Codes, Corp., Ann Arbor, MI) and aligned sequences to identify sequence polymorphisms. For each unique sequence, we used the Basic Local Alignment Search Tool (BLAST) to search against the entire nucleotide database (nr/nt) on GenBank for species identification [43]. Representative sequences were deposited in GenBank (KM281726-KM281801).

### Pollinator foraging behavior

To assess whether yeasts affected bumble bee pollinator foraging behavior, we conducted a behavioral assay in 2012 using experimental arrays of *Delphinium* in a portable greenhouse (WeatherPort Inc; Gunnison, CO, USA). Arrays were constructed using cut, virgin inflorescences set in green, translucent waterpics in a 4×3 grid (*N* = 12 stalks) with 15 cm spacing between pics. Newly cut, virgin inflorescences were used for each trial, with six inflorescences each treated at the whole-inflorescence level with either yeast-inoculated or control solutions.

In the yeast-inoculation treatment, we manipulated yeast density by adding 2 µl of yeast-inoculated 50% (w/w) sucrose solution (incubated for 5 days) to open flowers using a 25 µl Hamilton syringe (Hamilton Co., Reno, NV, USA). The yeast-inoculated solution was made with cells isolated from *Metschnikowia reukaufii*, a cosmopolitan, ascomycetous yeast of the *Metschnikowia* clade. This yeast was the only species found in our *D. barbeyi* nectar samples (see *Results*) and is also commonly associated with floral nectar and pollinators globally [6], [12], [14], [44]. Cells of an isolated *M. reukaufii* strain local to RMBL were obtained from cultures maintained on YM agar supplemented with chloramphenicol. Yeast-inoculated solutions were incubated for 5 days because this time period falls within an average flower's timespan [45], and the addition of 2 µl of sucrose solution falls within the range of lifetime floral nectar production and so can be experienced by foragers naturally [45], [46]. Flowers on control inflorescences received sterilized 50% (w/w) sucrose solution of the same volume, using a separate syringe to prevent cross-contamination. We wiped the syringe tips clean of pollen between plants using 70% ethanol, and care was taken to ensure that each nectar spur per flower received some solution. Because of the complex morphology of *Delphinium* flowers, we did not remove nectar from flowers prior to adding solutions to avoid damaging flowers. Because some “virgin” flowers can become colonized by yeasts through vectoring by other flower visitors, such as beetles (see Results), our control and yeast treatments may not reflect absence vs. presence of yeasts. Thus, our treatment applications reflect a dilution or augmentation of yeast cells, respectively, and prior research on a related species, *D. nuttallianum*, showed that diluting or augmenting yeast cells results in statistically significant low and high yeast cell densities that fall within the natural range of yeast cells exhibited in nature [25].

We used wild-caught queen *B. appositus* (*N* = 15) and *B. flavifrons* (*N* = 20). Bees were cooled on ice prior to foraging trials. Each trial consisted of an individual bee foraging on the array. The positions of yeast-inoculated or control treatments within the array were randomized for each bee. We monitored foraging decisions of each bee individually using hand-held voice recorders, noting the treatment identity of inflorescences and flowers visited, in addition to foraging time per flower (sec). After the foraging trials, bees were released at their collection location.

### Statistical analyses

All statistical analyses were performed using R version 3.0.2 [47]. To test whether pollinators may be responsible for vectoring yeasts, we compared yeast densities across all virgin (not exposed to pollinators prior to bagging) and open (exposed to pollinators prior to bagging) flowers sampled by fitting a linear mixed effects model using the *nlme* package with yeast density (log_10_(x+1)transformed) as the dependent variable and visitation status (open/virgin) as the explanatory variable. Since multiple open and virgin flowers were taken from the same plant, and few plants had both open and virgin flowers for comparison (*N* = 16), we pooled plants across sampling events (dates and sites) in the analysis. To control for this, we treated sampling date, site, and plant identity as random effects in our model, with plant nested within date, nested within site.

To test for differences in yeast cell density between sex phases of open flowers, and whether the potential differences between sex phases varied amongst sites, we fit a linear mixed effects model with sex-phase and site as fixed factors and their interaction. Plant identity and sampling date were included as random effects with plant identity nested within sampling date. Because we only measured yeast density in three sites, we included site as a fixed and not a random effect. We included plant identity to avoid pseudoreplication due to sampling multiple flowers from within the same plant. Cell density was log_10_(x+1) transformed prior to analysis.

To examine effects of nectar-inhabiting yeasts on pollinator foraging behavior, we fit separate linear mixed effects models for each component of foraging behavior (proportion of visits, proportion of flowers probed, and foraging time per flower) examined. For each forager, we calculated the proportion of total visits, mean proportion of flowers probed, and foraging time per flower for each respective nectar treatment. In our models, these behavior metrics served as response variables and nectar treatment (control or yeast), species identity (*B. appositus* or *B. flavifrons*), and their interaction were treated as fixed effects. In each model, forager identity was treated as a random effect to avoid pseudoreplication of behavior measurements within individuals.

## Results

### Density of yeasts

**Figure 1:**
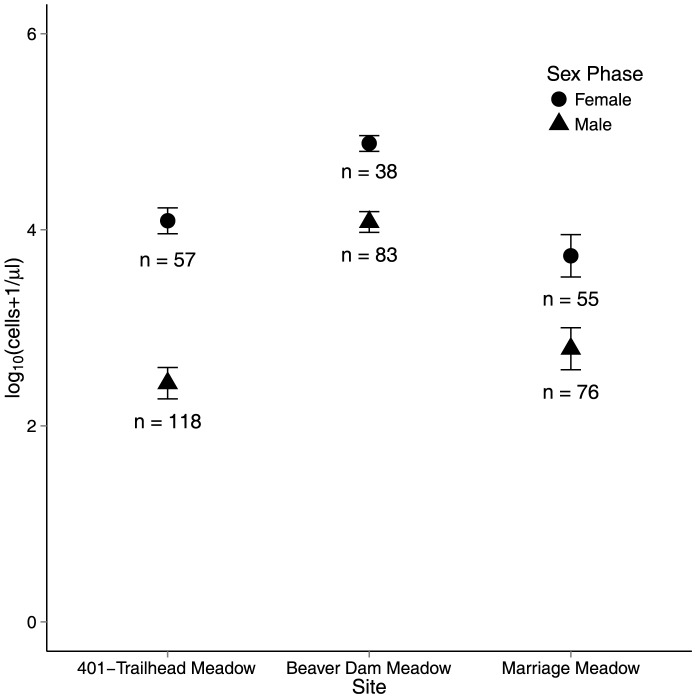
Yeast cell density (log_10_(x+1) transformed) in the nectar of *Delphinium barbeyi* flowers of different sex phase (male and female) in three populations. Symbols represent means and vertical segments are ±SE.

Yeast density differed significantly between virgin and open flowers examined (*F*_1,186_ = 132.61, *p*<0.0001). Only 11% of bagged virgin flowers contained yeasts, whereas 85% of open flowers that had been exposed to pollinators contained yeasts. Yeast density varied among floral sex phases (Fig. 1; *F*_1, 366_ = 97.5, *p*<0.0001), with female-phase flowers regularly harboring higher densities of yeasts in comparison to those in male-phase. The magnitude of this difference between floral-sex phases did not vary significantly across sites (site × sex-phase interaction: *F*_2,366_ = 2.87, *p* = 0.06). Moreover, we did not detect a significant main effect of site on yeast density (*F*_2,3_ = 1.42, *p* = 0.37). The proportion of *Delphinium* samples colonized by yeasts ranged from 77% at Beaver Dam to 67% at 401-Trailhead Meadow to 54% at Marriage Meadow (54%).

### Yeast identification

A total of 76 isolates were obtained from the 28 nectar samples cultured. All of the isolates were of a single species, the ascomycetous yeast *Metschnikowia reukaufii*.

### Pollinator foraging behavior

**Figure 2:**
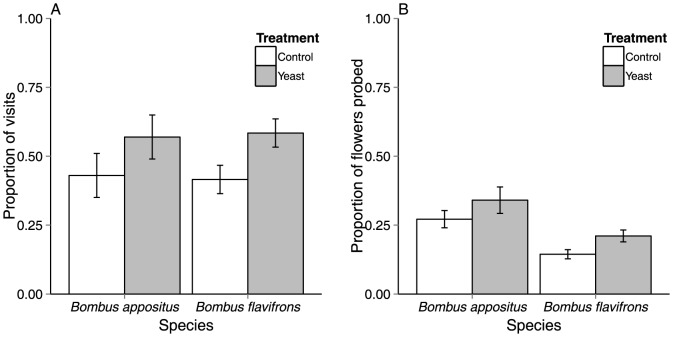
Mean (A) proportion of visits and (B) proportion of flowers probed per foraging bout within an inflorescence for *B. appositus* and *B. flavifrons* to control and yeast-treated *Delphinium barbeyi*. Bars represent means +SE.

Pollinators responded positively to the presence of yeasts. A significantly higher proportion of visits made by *Bombus* foragers were to yeast-treated inflorescences rather than to controls (Fig. 2A: *F*_1,30_ = 5.8, *p* = 0.02). This effect on foraging behavior was consistent across species, as we detected no significant yeast treatment by species interaction (*F*_1,30_ = 0.05, *p* = 0.82). Moreover, *Bombus* foragers probed significantly more flowers on yeast-treated inflorescences than controls (Fig. 2B: *F*_1,29_ = 5.85, *p* = 0.02). We also detected a significant differences between *Bombus* species in probing behavior, with *B. appositus* probing a greater proportion of flowers compared to *B. flavifrons* (*F*_1,33_ = 15.33, *p* = 0.0004). However, foraging time per flower was not affected by the presence of yeast (*F*_1,33_ = 0.009, *p* = 0.92), nor did pollinator species differ in foraging time (*F*_1,33_ = 0.016, *p* = 0.90). Pollinators spent (mean +SE) 10.63+0.4 sec on yeast-treated flowers and 10.76+0.5 sec on control flowers.

## Discussion

Plants participate in myriad interactions with microorganisms both above- and below-ground. Phyllosphere-inhabiting microorganisms are emerging as potentially significant interactors, with the ability to mediate the strength and nature of both antagonistic and mutualistic interactions in which plants participate [18], [24], [25], [48]. Here, we document that nectar-inhabiting yeasts, frequent colonists of floral nectar in *Delphinium* and other angiosperms, can significantly alter the foraging behavior of generalist bumble bee pollinators.

We detected a significant difference in both the frequency of occurrence and density of yeasts observed between virgin flowers and those open to visitation by larger pollinators, with a significantly lower proportion of virgin flowers colonized. This result suggests an important role of large pollinators as vectors for yeast dispersal and inoculation. All bagged flowers that we termed virgin, however, were not free of yeasts; yeasts were present in a small percentage (11%) of flowers bagged to exclude large pollinators. Dissection of these flowers for nectar removal revealed unidentified floricolous beetles (Phillips, *pers obs*). Nectar surveys of other systems have revealed that beetles can be an important source for yeast inoculation of floral nectar [49], [50]. The identity of the beetles in the *Delphinium* flowers and whether or not they can indeed vector yeast remains to be investigated. Nonetheless, our results point to the importance of larger floral visitors, such as bumble bees, hummingbirds, hawkmoths, and solitary bees, as the primary vectors of yeast to *Delphinium* flowers, as has been observed in other systems [28], [51].

Yeast frequency of occurrence and density varied significantly across the different spatio-temporal scales examined. Both the proportion of flowers colonized by yeasts and densities observed varied across the three *Delphinium* populations examined, with the Beaver Dam population having both the highest observed density and proportion of flowers colonized. These estimates of frequency of occurrence and density are similar to other population and community surveys [12], [15], [46], [52]. Such among-population variation in yeast dynamics may be a function of multiple processes. For example, differences in phenology among populations, which could influence the timing of interactions between plants and pollinators whom vectors yeasts, may serve as an important source of variation in yeast dynamics observed within and among populations. In addition, such differences may be a function of differences in the abiotic environment experienced by plant populations. For example, Golonka and Vilgalys [8] found evidence for a significant influence of water availability on yeast abundance. Variation in water availability, in addition to other abiotic factors, may influence both the availability and quality of habitat (i.e., nectar) available to yeasts [8]. Yeast density also varied with flower lifetime and its associated sex-phase transition, as yeast densities were significantly higher in female-phase flowers in comparison to those that were male-phase. This finding matches other surveys of protandrous, hermaphroditic systems [6], [22], where older, female-phase flowers typically harbor higher densities of yeast. Such differences are likely a function of both duration of exposure to visitation by pollinators who vector yeasts, thus increasing the probability of inoculation, and incubation time of the yeast in nectar.

The yeast community associated with *Delphinium* nectar was species-poor. Molecular analysis revealed a single species, the ascomycetous yeast *Metschnikowia reukaufii*, a cosmopolitan yeast that frequently colonizes floral nectar and has been observed in a number of floral systems [5], [12]. Nectar microbial communities are often species-poor, with species richness estimates for yeasts ranging from 1.2–1.7 [15], [52], [53]. In general, this low species richness of nectar yeast communities is likely a function of dispersal limitation [14], strong interspecific competition [22], and the selective environment imposed through the chemical makeup of nectar [10], [28]. *Delphinium* nectar communities are likely species-poor for at least two reasons. First, numerous floral-sphere yeasts have limited osmotolerance and are incapable of growing at sugar concentrations greater than 40% [10]. *Delphinium* has a mean nectar sugar concentration greater than that (44+3%, Irwin, *unpub data*), which likely precludes colonization by most nectar-inhabiting yeasts. *Metschnikowia reukaufii*, however, readily grows in nectar with sugar concentrations within this range [10]. Second, *Delphinium* has low concentrations of norditerpene alkaloids in its nectar [54]. It has been hypothesized that secondary compounds in nectar may serve an anti-microbial defensive function [9]; yet, evidence in support of this hypothesis has been mixed [10], [55]. Manson et al. [55] failed to detect an inhibitory effect of the alkaloid gelsemine in the nectar of *Gelsemium sempervirens* on the growth of a number of yeasts that colonize floral nectar. However, Pozo et al. [10] found that yeast growth was negatively affected by the alkaloids atropine and tropine found in the nectar of *Atropa baetica*. Given the well-known bioactivity of norditerpene alkaloids against vertebrate and invertebrate herbivores [56], [57], testing the effects of norditerpene alkaloids on nectar microbial communities warrants further investigation.

Yeasts elicited positive foraging responses by *Bombus* pollinators. *Bombus* visited and probed more yeast-inoculated flowers in comparison to controls, suggesting that *Bombus* may prefer yeasts or traits modified by their activity. This finding matches recent studies documenting bumble bee preference of yeasts and/or yeast-modified nectar. Both Herrera et al. [24] and Schaeffer and Irwin [25] found that pollinators removed significantly more nectar from flowers inoculated with yeasts, and the primary pollinators in both studies were *Bombus*. The proximate cues and mechanisms driving pollinator preference for yeast-inoculated flowers of *Delphinium* are unknown; however, we hypothesize that at least two mechanisms, which are not mutually exclusive, may be involved. First, yeasts may play an important role in honest signaling of nectar presence through the production of volatiles during fermentation [17], [20], [58]. Second, changes in amino acid, vitamin, or other metabolite availability as a consequence of yeast metabolism may be driving changes in pollinator foraging decisions [24]. For instance, shifts in amino acid concentration or composition as a consequence of yeast metabolism may affect nectar palatability [22]. Careful dissection of the effects of yeasts on nectar traits and their relative role in driving observed patterns of plant-pollinator interactions await experimentation.

Although our study highlights the potential for yeasts to mediate plant-pollinator interactions, three caveats are important to consider when interpreting our results. First, our study only examined the potential influence of one nectar-inhabiting microorganism, the cosmopolitan yeast *M. reukaufii*, on the behavior of *Bombus* pollinators. This yeast may not be the only nectar-microbial colonist. Recent surveys have indicated that bacteria can also frequently colonize floral nectar [7], [18], [59], with the potential to elicit negative foraging responses by a diversity of pollinators [18], [26], [60]. Moreover, our use of culture media (i.e., YM) and selection of only one morphologically distinct colony per sample for sequencing may have made our estimate of fungal diversity conservative. Alternative culturing methods may reveal additional fungal nectar colonists with similar or opposing effects on *Bombus* behavior. Second, in addition to only testing for effects of one microorganism, we only examined the foraging response of one guild of pollinators, bumble bees. *Delphinium* is frequently utilized as a nectar resource by hummingbirds, hawkmoths, and other floral visitors [27], whose energetic demands differ in comparison to bumble bees [61]. And third, though yeast frequency of occurrence and density varied spatio-temporally, the mean densities observed at each site were all dense enough to potentially elicit foraging responses by pollinators [18], [24], [25]. Thus, the ecological and evolutionary significance of such variation remains unclear. Future research should consider both whether pollinator responses to microorganisms are density-dependent [60] and the consequences of such responses at both spatial and temporal scales.

Given well-known effects of pollinator foraging behavior on plant fitness, our results suggest that nectar yeasts have the potential to indirectly alter pollen transfer dynamics and plant fitness mediated through changes in pollinator foraging [23]–[25]. Bumble bees foraging on *Delphinium* tend to visit in a bottom-up fashion, starting at flowers at the base of an inflorescence and working their way up flowers on a stalk, before switching to other stalks within a plant [27], [46]. In so doing, bumble bees encounter female-phase flowers at the bottom of the inflorescence first before male-phase flowers at the top, where they remove pollen and export it to other stalks or plants. Increased movements within a plant by pollinators seeking yeasts may affect the magnitude of geitonogamous pollination, with important consequences for patterns of plant mating and reproduction in this self-compatible species. Future research investigating the benefits of nectar yeasts on increased per-flower visitation vs. the costs of potential geitonogamous pollen transfer will yield additional ecological insights.
